# Electrophoretic Deposition of Layer-by-Layer Unsheathed Carbon Nanotubes—A Step Towards Steerable Surface Roughness and Wettability

**DOI:** 10.3390/ma13030595

**Published:** 2020-01-28

**Authors:** Emil Korczeniewski, Monika Zięba, Wojciech Zięba, Anna Kolanowska, Paulina Bolibok, Piotr Kowalczyk, Agata Wiertel-Pochopień, Jan Zawała, Sławomir Boncel, Artur P. Terzyk

**Affiliations:** 1 Faculty of Chemistry, Physicochemistry of Carbon Materials Research Group, Nicolaus Copernicus University in Toruń, Gagarin Street 7, 87-100 Toruń, Poland; e.korczeniewski@umk.pl (E.K.); monikaventabal@gmail.com (M.Z.); wuzet95@gmail.com (W.Z.); pbolibok@umk.pl (P.B.); 2 Department of Organic Chemistry, Bioorganic Chemistry and Biotechnology, Silesian University of Technology, Krzywoustego 4, 44-100 Gliwice, Poland; anna.kolanowska@polsl.pl (A.K.); slawomir.boncel@polsl.pl (S.B.); 3 College of Science, Health, Engineering and Education, Murdoch University, Perth, WA 6150, Australia; kowalczyk.piotr@wp.pl; 4 Jerzy Haber Institute of Catalysis and Surface Chemistry Polish Academy of Sciences, ul. Niezapominajek 8, 30-239 Kraków, Poland; ncwierte@cyfronet.pl (A.W.-P.); nczawala@cyf-kr.edu.pl (J.Z.)

**Keywords:** carbon nanotubes, electrophoretic deposition, roughness, wetting

## Abstract

It is well known that carbon nanotube (CNT) oxidation (usually with concentrated HNO_3_) is a major step before the electrophoretic deposition (EPD). However, the recent discovery of the “onion effect” proves that multiwalled carbon nanotubes are not only oxidized, but a simultaneous unsheathing process occurs. We present the first report concerning the influence of unsheathing on the properties of the thus-formed CNT surface layer. In our study we examine how the process of gradual oxidation/unsheathing of a series of multiwalled carbon nanotubes (MWCNTs) influences the morphology of the surface formed via EPD. Taking a series of well-characterized and gradually oxidized/unsheathing Nanocyl™ MWCNTs and performing EPD on a carbon fiber surface, we analyzed the morphology and wettability of the CNT surfaces. Our results show that the water contact angle could be gradually changed in a wide range (125–163°) and the major property determining its value was the diameter of aggregates formed before the deposition process in the solvent. Based on the obtained results we determined the parameters having a crucial influence on the morphology of created layers. Our results shed new light on the deposition mechanism and enable the preparation of surfaces with steerable roughness and wettability.

## 1. Introduction

Electrophoretic deposition (EPD) is one of the most popular and promising methods for the preparation of different nanostructured surfaces and devices [1,2], especially carbon nanotube (CNT)-reinforced fibers [3,4,5]. It was shown [6] that the ultrasonically assisted (i.e., ultrasound is applied throughout the deposition process) EPD of CNTs and nanotube forests [7] on carbon fibers (CFs) or on glass [8,9,10] improved the mechanical properties, interfacial shear strength, and friction resistance [11] of the final fibrous hierarchical composites. CNTs have been applied to improve the ablation resistance of carbon–carbon composites working at very high temperatures [12]. Here other techniques, such as the catalytic growth of CNTs, are not applicable due to the co-introduction of metal residues. Thus, EPD can be successfully applied [12]. Additionally, an improvement of the roughness and surface polarity of CNTs by EPD can lead to wettability improvement [13]. For example, Li et al. [14] studied the wettability of CNT-coated CFs and noticed the decrease in the water contact angle (WCA) with the increasing roughness caused by CNT electrodeposition. Jiang et al. [15] reported the WCA on CF to be equal to ca. 76°, which decreased to 57–51° after nanotubes deposition depending on the application of ultrasonication during the process.

Although EPD was recently supported by the application of laser-induced welding [16] (enhancing the overall mechanical properties of the CNT coating), this method has generally been applied in a typical form. Thus, the deposition of vertically aligned CNTs [17,18] or CNTs for the production of buckypapers [19,20,21,22]; electron field-emission devices [23,24,25]; microcapacitors [26]; fibers applied for solid-phase microextraction [27]; patterned [28,29,30], templated [31], porous or radially grafted nanotube structures [32]; electrodes for solar cells [33]; thermal absorbents [34]; vertically aligned CNT electrodes [35,36]; sensors [37] nanotube–polymer composites [38,39]; spectrally selective absorbers [40]; touch screen panels [41]; topographically designed surfaces [42]; and biocoatings [43,44] have been reported, among others.

One of the most important advantages offered by EPD is the possibility of forming thin homogeneous films, and this plays an important role in the application of carbon materials in solar cells, field-emission devices, coatings (including biocoatings [43,45,46]), etc. [47]. For example, electrophoretic coating of CNTs by dielectric-constant ceramic compounds was shown to be more effective than classical CVD during the construction of three-dimensional capacitors [48]. It was also proved that the film thickness depends (among other things) on the type of applied solvent [49]. It was explained [1,50] that the composition of solvent changes the nanoarchitecture of the forming layer. Moreover, EPD offers the smoothest deposits among other widely applied methods [51] for the nanotube films. This advantage can be important in different biomedical applications of CNTs. For example, Benko et al. [52] studied the deposition of CNTs on titania, and the purpose of their study was to verify the influence of the obtained layers on cell growth. They found the presence of surface oxygen groups on the CNT surfaces, and the favorable conditions for the growth of osteoblasts.

From the analysis of the literature data cited above, it can be concluded that the number of papers describing the EPD of CNTs is enormous, and CNTs are very important materials applied for deposition due mainly to their electric and mechanical properties. However, little is known about the details of the EPD mechanism if nanomaterials, especially CNTs, are deposited. This is why we decided to perform our study using CNTs with a controlled number of walls and originated from the same initial material. It is well known that the oxidation of nanotubes (usually with concentrated HNO_3_) is applied as the initial stage for the preparation of CNTs before the EPD process [47]. However, recently published reports shed new light on the influence of nanotube oxidation on CNTs’ morphology. Namely, it was proved that the oxidation of CNTs led to simultaneous unsheathing [53]. This so-called “onion effect” was confirmed by our groups for different oxidizing agents and different CNTs. Additionally, the kinetics of the unsheathing process was determined [54].

Layer-by-layer (precisely, wall-by-wall) nanotube unsheathing—also called the “onion effect”, recently studied by González et al. [53]—is a process of the stepwise, non-monotonic and quasi-periodic functionalization (mainly with carboxylic -COOH and hydroxyl -OH groups) and defunctionalization of multi-wall carbon nanotubes. This effect is based on the fact that as the functionalization of an MWCNT containing *n* walls proceeds (and hence as the outermost nanotube wall gains more functional groups), at higher functionalization levels this wall becomes more and more loosely bound, eventually being shed. At the same time, the wall lying just below the outer wall (*n −* 1) is revealed (simultaneously, an excessive concentration of functional groups in close proximity—forming highly reactive hot spots—leads to nanotube cutting). From this point, the oxidation starts for the *n -* 1 nanotube wall, continues for deeper-lying walls, and is observed until all the walls are peeled off (i.e., until the total oxidation of the carbon skeleton to thermodynamically stable carbon dioxide and water). This phenomenon is of particular importance since carboxyl-functionalized wall is hydrophilic while the non-functionalized wall is hydrophobic. Hence, the conditions of functionalization must be rigorously controlled. Moreover, the functionalization must be strictly monitored both qualitatively and quantitatively. The effect was also recently confirmed and further studied by Kolanowska et al. [54]. As the authors demonstrated for progressively functionalized and quenched MWCNTs over the oxidation under harsh conditions (boiling in nitrating mixture of concentrated H_2_SO_4_ and concentrated HNO_3_, *v/v* = 3/1), inter alia by counting the nanotube walls, titration of COOH groups, and quantitative TGA for the products, nanotubes could indeed be identified as alternatively functionalized and non-functionalized. Notably, here the “onion effect” was cross-verified by measuring zeta-potential in the products of MWCNT oxidation over time. Therefore, higher degrees of functionalization (as confirmed by Boehm titration) with surface carboxylic groups—dissociable in water to negatively charged carboxylate (COO^–^) and protons—fully corresponded with a more negative zeta potential (e.g., [55]). The latter one is very important value in surface chemistry (e.g., [56,57]).

Since there are no reports showing how the morphology and wettability of a CF surface changes after the EPD of CNTs containing a progressively decreasing number of walls, we decided to examine this phenomenon in the current report. Thus, the aim of this study was not only to obtain a series of materials containing progressively unsheathed CNTs deposited on a CF surface (and to study the morphology of agglomerates) but simultaneously, knowing the characteristics of CNTs, we addressed which parameters determine the mechanism of deposition. To our best knowledge, since the “onion effect” has been discovered only recently, this is the first report concerning the influence of unsheathing on the properties of thus-prepared CNTs surface layers.

## 2. Materials and Methods

As a starting material we applied commercially available multiwalled CNTs NC7000™ (d = 9.6 nm, l = 1.5 μm, 90% purity) from Nanocyl™ (Nanocyl™, Sambreville, Belgium). They were oxidized and unsheathed using nitric (65%, pure p.a.) and sulfuric acids (98%, pure p.a., Chempur, Piekary Śląskie, Poland), according to the procedure described previously [54]. The following oxidation times were applied (the samples are labelled as follows): 10, 15, 30, 75, and 90 min. CNTs were characterized by the Boehm titration and detailed analysis leading to the number of walls, according to the procedure described previously [54].

Nanomaterials used for the deposition were characterized by: transmission electron microscopy (TEM) (FEI, Tecnai F20 X-Twin, Particulate Systems, Norcross, GA, USA), N_2_ adsorption–desorption (Gemini, Micromeritics, Particulate Systems, Norcross, GA, USA), DLS, and ζ-potential (25 °C, Particulate Systems, NanoPlus HD, Micromeritics, Particulate Systems, Norcross, GA, USA) using solutions having concentration of 0.1 mg/mL (volume 20 mL) after 5 min sonication (60 W, BANDELIN, Sonoplus HD 4100, BANDELIN, Berlin, Germany).

The EPD processes were carried out on the surface of CFs purchased from the HP-textiles GmbH (art. no. HP-T193C, HP-Textiles GmbH, Schapen, Germany) purified by heating at 500 °C in N_2_ (95%) for 3 h, between previously prepared carbon papers for the polymer-coating sorption, and removal of the other impurities. Suspensions of CNTs 0.05 mg/mL (200 mL) in isopropanol (99.7%, pure p.a., Chempur, Piekary Śląskie, Poland) were prepared by sonication two times for 5 min (60 W) with 2 min breaks to prevent overheating. After 10 min of sedimentation, suspensions were used for the deposition.

The EPD processes were carried out in isopropanol (99.7%, pure p.a., Chempur, Piekary Śląskie, Poland) under 300 V/cm for 10 min. Applied potential and time of deposition were used to maximize the volume of the deposited coating. As the electrodes, CFs and nickel were applied. Due to negative ζ-potential values in isopropanol (between −12 and −36 mV—see Table 1) of CNTs, the EPD process was carried out on the anode. After deposition the samples were washed out with isopropanol and dried at 70 °C in air for 5 min.

The surfaces of initial and CNT-coated CFs were characterized by a scanning electron microscope (SEM) (Quanta 3D FEG, Thermo Fisher Scientific, Brno-Černovice, Czech Republic). Roughness factors were calculated (program NanoScope Analysis, Bruker, Bremen, Germany) based on atomic force microscope analysis (MultiMode with scanner type E, tapping mode, Veeco Metrology, Santa Barbara, CA, USA). During this analysis, from five to ten areas were checked in different places on the surface and then the roughness was calculated. Just before the WCA measurements, the samples were desorbed at 130 °C in air for 20 h and slowly cooled down in a glass container to avoid the adsorption of impurities.

WCA was measured (at 25 °C) six times for each sample using a homemade goniometer having a fixed-focus lens (as reported previously [58]) with a camera Grasshopher3 GS3-U3-32S4C-C, 3.2 Mpx. The error of WCA was different for different samples but not larger than ±3°. To define the changes in the surface ζ-potential of materials, “flat surface measurements” of ζ-potential were conducted, using the “flat surface” cell (water at 25 °C, Micromeritics, Particulate Systems, Norcross, GA, USA) and the method described by Corbett et al. [59]. To do this, we used the sample monitor solution with tracer particles produced by Otsuka Electronic Co. (Osaka, Japan). The tube diameters were determined by a statistical TEM analysis (F20X-TWIN, FEI-Tecnai, Thermo Fisher Scientific, Brno-Černovice, Czech Republic) of 50 individual and distinguishable (i.e., visibly separable from one another, measured one-by-one) nanotubes for a given batch.

## 3. Results

### 3.1. Characteristics of CNTs before the EPD Process

**Figure 1:**
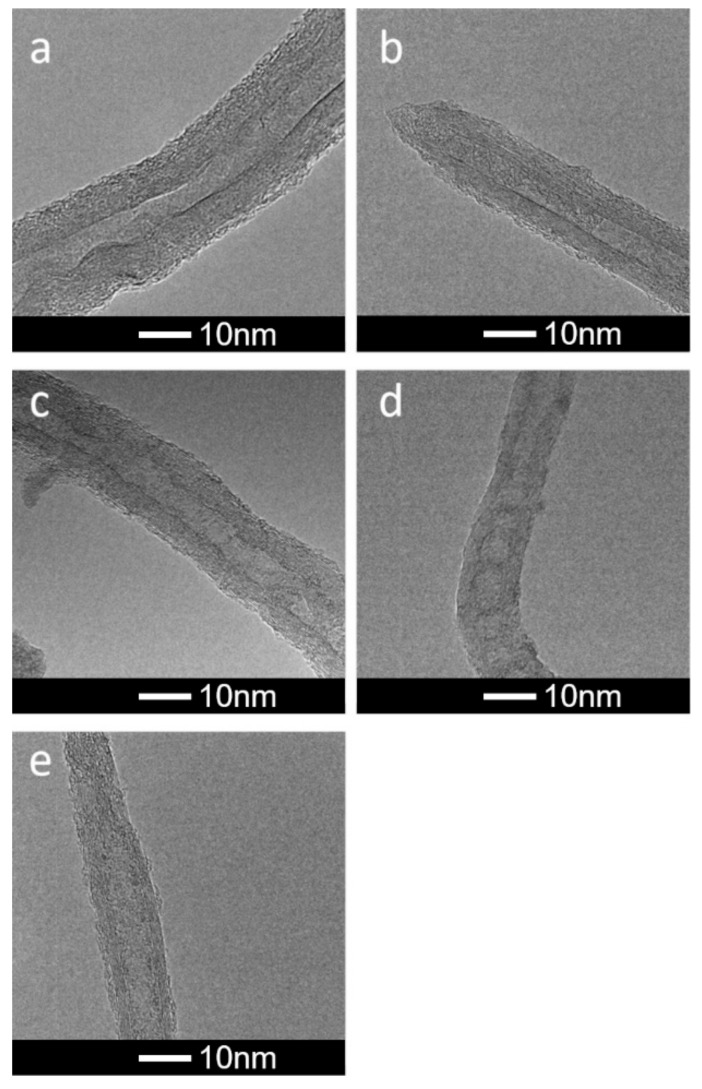
HRTEM images showing the influence of oxidation/unsheathing on the thickness of CNTs applied during the EPD process for oxidation times: (**a**) 10, (**b**) 15, (**c**) 30, (**d**) 75, (**e**) 90 min.

Table 1 collects selected results of CNT characterization before and after the EPD process. As one can see, with the time of oxidation the average number of walls (confirmed by the TEM images—see Figure 1) and the values of the Brunauer-Emmett-Teller (BET) surface areas decreased. The concentration of surface carboxylic groups changed irregularly, and this was caused by two effects. With the oxidation time the number of surface carboxylic groups increases (while the concentration of hydroxyl groups is almost constant), but afterwards, the process of unsheathing occurs. Since COOH groups are bonded mainly to the external CNT walls, this effect is accompanied by a decrease in the concentration of surface carboxylic groups. Further oxidation produces new COOH groups, etc. Since the number of walls (see Figure 1) and the tube radius decrease as the oxidation time progresses, one should expect the rise in the values of specific surface areas, as proved by the GCMC simulation data [60] and geometric calculations [61]. However, this was not the case (Table 1), and it can be straightforwardly explained by the well-known phenomenon of nanotube bundle formation [61].

### 3.2. Characteristics of CNTs after the EPD Process

**Figure 2:**
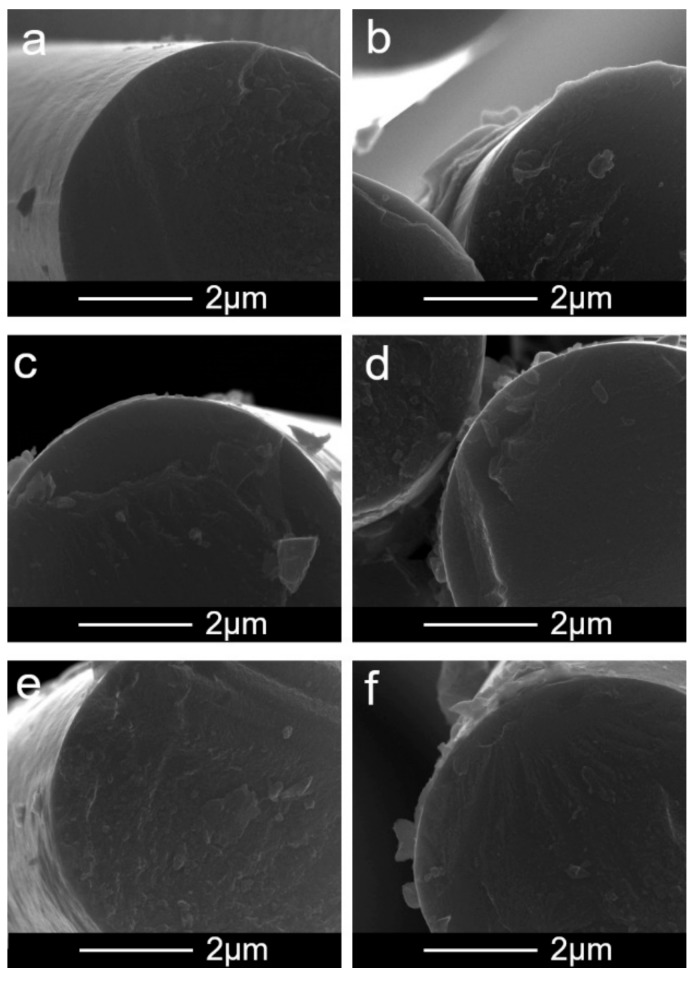
SEM images showing the influence of oxidation/unsheathing on the morphology of CNT layers formed after the EPD process: (**a**) CF before the EPD, (**b**) oxidation time 10 min, (**c**) 15 min, (**d**) 30 min, (**e**) 75 min, (**f**) 90 min.

**Figure 3:**
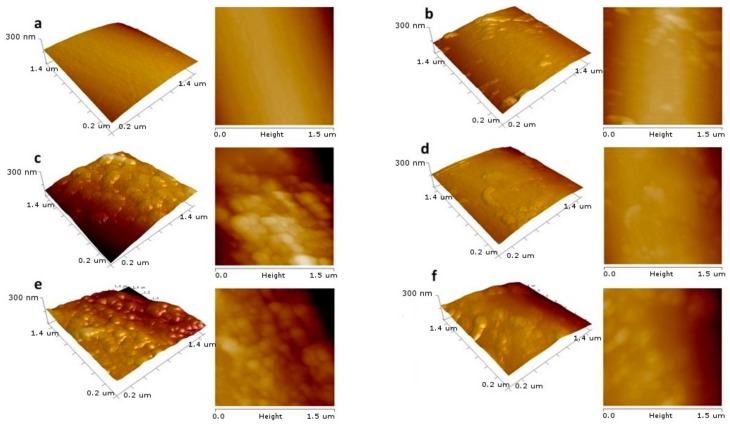
2D and 3D height images of obtained surfaces from the AFM tapping-mode analysis, showing the influence of oxidation/unsheathing on the morphology of CNT layers created after the EPD process: (**a**) CF before the EPD, (**b**) oxidation time 10 min, (**c**) 15 min, (**d**) 30 min, (**e**) 75 min, (**f**) 90 min.

Figure 2 collects the SEM images showing the layer of deposited CNTs on a CF. One can observe that the layer was relatively homogeneous. However, there were differences in roughness between the surfaces. This is confirmed by the results collected in Figure 3 showing the AFM-based surface morphology.

**Figure 4:**
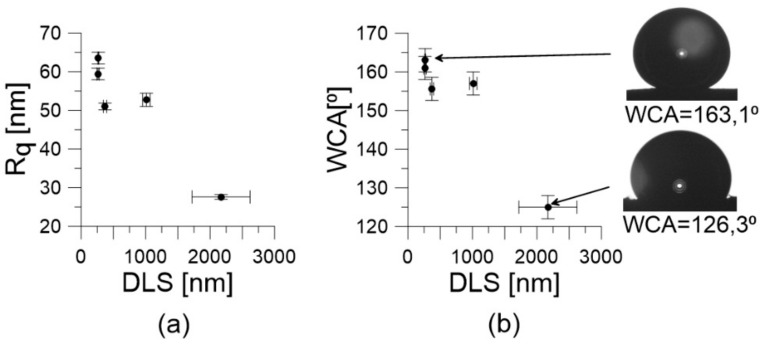
(**a**) The correlation between roughness factor (Rq) and DLS diameter; (**b**) The relation between water contact angle (WCA) and DLS diameter.

We did not observe a correlation between the roughness factor (Rq) values, the CNTs’ diameter, and the BET surface areas. Moreover, the surface ζ-potential of materials obtained after the EPD process (Table 1) was practically the same for all obtained samples. However, from the results collected in Figure 4a, one can conclude that Rq values depended on the DLS diameters of nanotubes measured in isopropanol (Table 1)—that is, the larger the aggregate diameter, the smaller the Rq value was.

**Figure 5:**
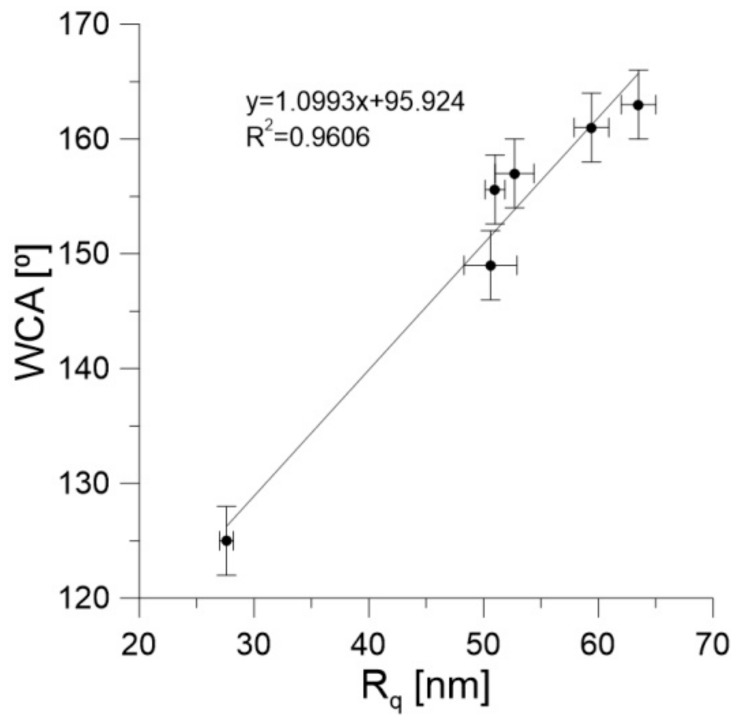
WCA dependence on the Rq, together with the results of fitting to the straight line. Note that for Rq close to zero the WCA was ca. 96°.

In Figure 4b we show the relation between the DLS diameter and the WCA. As one can conclude, the WCA values decreased with increasing aggregate diameter. This finally leads to the correlation between WCA and the Rq values shown in Figure 5. It can be seen that the WCA values increased with increasing Rq. Moreover, the range of WCA was relatively wide (125–163°).

## 4. Discussion

Based on the obtained results, we can estimate the important stages of the EPD process. It is well known from the Hamaker equation [62] that ζ-potential of a colloid is one of the most important factors determining the EPD and the thickness of deposited layer. In the case of the studied systems, we notice that the ζ-potential of CNTs in isopropanol mainly influenced the kinetics of the deposition process. Most importantly, we did not observe correlations between the ζ-potential of unsheathed CNTs and the properties of the obtained surfaces. This does not agree with some results suggesting that the value of ζ-potential of particles can influence the roughness of surfaces obtained after coagulation [63]. Our results clearly indicate that the diameter of aggregates in isopropanol is a crucial factor determining the morphology of the CNT surfaces. Since the values of surface ζ-potential were similar and close to zero (Table 1), one can conclude that all thus-formed surfaces bore negligibly dissociated functional groups, which corresponds to poor dissociation of carboxylic acids in a base much weaker than water (i.e., isopropanol). Predominantly, however, it is reasonable to assume that the groups (bonded mainly to the edges and defects of tube walls) are involved in the formation of the three-dimensional network of hydrogen bonds during the aggregation in isopropanol. Additionally, we did not observe a correlation between the concentration of CNT surface groups, the number of walls (and the other parameters characterizing unsheathed nanotubes), and the DLS diameter in isopropanol. Thus, to fully control the morphology of a surface, it is crucial to find this relationship. This necessity requires further studies in different solvents, and these results will be reported in the future work.

The observed WCA values changed in a relatively wide range (Figure 4b and Figure 5) and all surfaces were hydrophobic. This additionally confirms the absence of surface-oxygen-containing groups, and supports the mechanism suggested above. However, considering that some authors have observed a decrease in WCA after the deposition of nanotubes [14,15], it is possible that by using the same nanotube samples one could fully control a very broad range of WCA values. This means that nanotubes are very promising materials for the preparation of surfaces with steerable wettability in a very wide range of WCAs (from hydrophobic to hydrophilic).

One can also observe an excellent correlation between the Rq and the WCA (Figure 5). Moreover, our experimental data extrapolated to Rq = 0 (i.e., for a perfectly smooth surface) leads to a WCA of 95.90°. This is in good agreement with recently reported WCA values for water on graphene, being in the range of 92–98° [64]. The rise in the WCA with Rq is caused by the Cassie–Baxter effect [65]. However, the most important is that the increase in WCA observed in Figure 5 is not caused directly by the decreasing number of nanotube walls (as was expected) but by the increasing tendency of nanotubes to form aggregates in isopropanol. Nevertheless, this tendency is a complicated and still unknown function of solvent, temperature, and nanotube properties, as well as the conditions of coating preparation [66].

## 5. Conclusions

Our results demonstrate that the WCA in CNT-coated CFs can be gradually changed and the major property determining its value is the diameter of the aggregates formed before the deposition process in a solvent. The zeta potential of the initial nanotubes only influences the kinetics of deposition and has no influence on the morphology of the coatings. We believe that the presented results are a step towards the synthesis of CNT surfaces with controlled wettability. However, further studies are necessary for the full control of surface morphology, and the results will be reported in upcoming works.

## Figures and Tables

**Table 1:** Characteristics of carbon nanotubes (CNTs) before and after the electrophoretic deposition (EPD) process. CF: carbon fiber.

Materials before deposition						
Oxidation time (min)	10	15	30	75	90	
No. of walls (*n*)	12.5 ± 1.4	12.2 ± 1.8	11.3 ± 1.8	8.8 ± 1.2	8.1 ± 1.2	
Diameter (nm)	18.9	14.1	15.4	11.1	10.1	
COOH content (mmol/g)	2.8	4.0	2.7	4.2	2.7	
BET (m^2^/g)	180	169	137	149	55	
ζ–potential (mV)	35.66 ± 2.65	−14.76 ± 5.23	−12.46 ± 5.15	29.28 ± 6.76	36.06 ± 6.21	
DLS diameter (nm)	2170 ± 450	267.3 ± 8.7	1012 ± 58	263 ± 19	371 ± 26	
**Materials after deposition**						
Oxidation time (min)	10	15	30	75	90	Initial CF
Surface ζ-potential (mV)	0.31 ± 0.42	−0.07 ± 0.33	−0.38 ± 1.13	−0.64 ± 0.9	0.39 ± 0.97	0.32 ± 0.40
